# Factors contributing to delayed diagnosis of cervical cancer in human immunodeficiency virus-positive women

**DOI:** 10.4102/safp.v67i1.6221

**Published:** 2025-12-08

**Authors:** Frenzar M. Tshiruruvhela, Mbuyisa J. Makhubu, Gert J.O. Marincowitz, Clara Marincowitz

**Affiliations:** 1 Department of Family Medicine, Faculty of Health Sciences, University of Limpopo, Mankweng, South Africa; 2 Department of Biological Sciences, Faculty of Science, University of Cape Town, Cape Town, South Africa

**Keywords:** PAP smear, papanicolaou smear, cervical cancer screening, HIV-positive women, cervical cancer

## Abstract

**Background:**

Cervical cancer is more prevalent in human immunodeficiency virus (HIV)-positive women and is frequently diagnosed in an advanced stage. This study sought to understand factors contributing to the delayed diagnosis of cervical cancer in HIV-positive women at Mokopane Hospital in the Limpopo Province of South Africa.

**Methods:**

A qualitative phenomenological study was conducted using individual interviews among purposively sampled HIV-positive women who were diagnosed with cervical cancer. Interviews were recorded, transcribed verbatim, translated and analysed thematically.

**Results:**

Four major themes emerged from the study, providing possible explanations for the delay in cervical cancer diagnosis. Firstly, women believed that Papanicolaou (PAP) smears are diagnostic and should be performed when symptoms appear, rather than for screening. Secondly, their readiness, fear and embarrassment to do the procedure delayed them from having a PAP smear. Thirdly, consulting traditional healers initially, also caused delays. Fourthly, a lack of equipment, inadequate follow-up, health workers’ low index of suspicion and unwillingness to do the procedure further delayed diagnosis.

**Conclusion:**

Human immunodeficiency virus-positive women have an inadequate understanding of cervical cancer screening and its importance. Patient education and health worker training are urgently needed to improve the screening. To counteract delays in screening, adequate staffing, regular maintenance and availability of equipment are vital for improved care for HIV-positive women.

**Contribution:**

This study highlights how a poor understanding of cervical cancer screening, patients’ readiness, fear and embarrassment to do the procedure, as well as inadequate equipment and poorly motivated health workers, all contribute to the delayed cervical cancer diagnosis in HIV-positive women.

## Introduction

Human immunodeficiency virus (HIV)-positive women have a six-fold increase in risk for developing cervical cancer compared to women who are HIV-negative.^1^ In southern Africa, 63.8% of women with cervical cancer are living with HIV, while globally, this figure is only 5.8%.^1^ It is estimated that up to 91% of all cases of women living with HIV and cervical cancer live in southern (64%) and eastern Africa (27%).^1^

According to the Cancer Report by Statistics SA, between 2008 and 2019^2^ the highest incidence of cervical cancer was found among black African women, a finding that was attributed to the high burden of HIV and human papillomavirus (HPV) infections in this population.^3^ This co-occurrence is problematic, as HIV exacerbates HPV. Human immunodeficiency virus contributes to the depletion of pro-inflammatory and anti-inflammatory pathways in HIV-positive women’s cervical mucosa, meaning they are less likely to clear an HPV infection. The latter increases their risk for cervical cancer.^4^ However, when detected early through screening programmes, cervical cancer is curable.

Unfortunately, socio-economic inequalities make screening less accessible to lower-income patients in sub-Saharan Africa.^5,6,7^

The national guidelines for cervical screening in South Africa^8^ recommend that, upon an HIV-positive diagnosis, all women should receive a PAP smear. Thereafter, this screening should be repeated every 3 years if cytology is negative. However, research conducted in KwaZulu-Natal reported that only 39.2 % of HIV-positive women had ever received a PAP smear.^9^ A similar notion was found in Limpopo province; however, this was a qualitative study and more research is needed.^10^ The reasons for failure to adhere to the national guidelines are unclear. International Agency for Research on Cancer concluded that there is sufficient evidence that cervical cancer screening can reduce cervical cancer mortality by 80% or more among screened women,^11^ making it crucial to understand inadequate cervical cancer screening practices. Therefore, our study aimed to investigate the factors contributing to a delayed diagnosis of cervical cancer in HIV-positive women at a district hospital in Limpopo province, South Africa.

## Research methods and design

A qualitative study was conducted at Mokopane Hospital. This hospital is situated near Mokopane town and is the referral centre for the Waterberg health district of Limpopo province in South Africa, serving a population of more than one million, predominantly rural Sepedi-speaking people.^2^

All 20 patients on the database in the gynaecology unit at Mokopane hospital who were HIV-positive and had cervical cancer stage 3 or stage 4 were approached to participate in the study. However, 2 patients were too sick to participate in the interviews, 5 patients died before the interview date because of complications of their disease, and 3 were unavailable for the interviews. The remaining 10 selected patients took part in the research.

Free attitude, non-directive interviews were conducted between June and August 2023. The initial exploratory question was: ‘What do you think are the factors that contributed to you being diagnosed late with cervical cancer?’ Further probing was carried out with three open-ended questions:

- What do you think about awareness of cervical cancer amongst women living with HIV?
- Explain what you think happened at the clinic or hospital that could have led to your delayed diagnosis of cervical cancer?
- What hindered you from seeking help when you experienced symptoms of cervical cancer?

Interviews were performedin a private room, where no interruptions were expected. An experienced research assistant conducted the interviews with the researcher present, taking field notes. The interviews were conducted in the language that participants were comfortable in, either English or Sepedi. The average duration of the interviews was 20 min. All interviews were audio-recorded. Demographic information, including the symptoms before their diagnosis of cervical cancer, was collected as an introduction to the interviews.

All the recorded interviews were transcribed verbatim by the research assistant, and the transcriptions were checked by the researcher for correctness. The transcribed interviews were then translated into English by a language expert and rechecked by the researcher. The data were analysed using interpretative content analysis. Familiarisation with the content of the interviews started during the interview, when the researcher was taking field notes. The researcher and the research assistant further familiarised themselves with the data by reading the transcripts and listening to the audiotapes several times. Key ideas and recurrent themes were noticed as they emerged. The thematic content analysis was guided by the five stages of thematic analysis, namely familiarisation, theme identification, indexing, charting, mapping and interpretation.^12^ The data were explored for common themes by creating a list of codes after the familiarisation process. These codes were then organised into categories. The final identified themes were then confirmed by using the cut-and-paste manual qualitative analysis method.^11^ During the interpretation stage, themes were arranged into a schematic model. The model represents the factors influencing late presentation with cervical cancer at the research site and demonstrates the interrelatedness between the themes.

Active attempts were made to ensure the trustworthiness of the study. Credibility, transferability, dependability and confirmability were enhanced with the following steps: prolonged engagement with the data, checking the transcription and emerging themes with the participants, triangulating the findings between the researcher and research assistant, and review by the research supervisor.

### Ethical considerations

Ethical clearance was granted by the University of Limpopo, Turfloop Research and Ethics Committee on 22 August 2022. The ethical clearance number is TREC/344/2022: PG. Permission to conduct the study was obtained from the Department of Health (LP2023-05-010), Waterberg Health District Office and the Chief Executive Officer (CEO) of Mokopane Hospital. Informed consent was obtained from all participants before their interviews.

## Results

All 10 participants were Sepedi-speaking black South Africans from Mokopane, with ages ranging from 39-50-years-old. Most of the participants had secondary education (*n* = 6), were unemployed (*n* = 7) and single (*n* = 7) (Table 1). One participant was in a cohabiting relationship, and 2 participants were married. Participants’ main presenting symptoms were menorrhagia, post-coital bleeding and pain (Table 1).

**TABLE 1:** Description of participants.

Participant number	Age (years)	Educational level	Marital status	Employment status	Year HIV was diagnosed	Initial cervical cancer symptom
1	40	Secondary	Not married	Unemployed	2010	Blisters on the vagina
2	59	Primary	Not married	Unemployed	2016	Pelvic pain
3	57	Secondary	Cohabitating	Employed	2011	Menorrhagia
4	42	Tertiary	Not married	Unemployed	2011	Fatigue, abnormal uterine bleeding and pelvic pain
5	48	Primary	Not married	Unemployed	2008	Menorrhagia and pelvic pain
6	46	Secondary	Married	Unemployed	2007	Pain and post-coital bleeding
7	49	Secondary	Not married	Employed	2005	Menorrhagia
8	45	Tertiary	Not married	Employed	2017	Post-coital bleeding and abnormal uterine bleeding
9	49	Secondary	Married	Unemployed	2021	Menorrhagia
10	39	Secondary	Married	Unemployed	2006	Abnormal vaginal discharge

HIV, human immunodeficiency virus; PAP, Papanicolaou.

From the interviews, we identified four main themes that contributed to the delay in diagnosis of cervical cancer in these 10 HIV-positive women, namely: (1) patients’ perceptions and knowledge of the cervical cancer screening process; (2) their fears and beliefs; (3) health systems limitations; and (4) healthcare worker knowledge, attitudes and practices.

**FIGURE 1:**
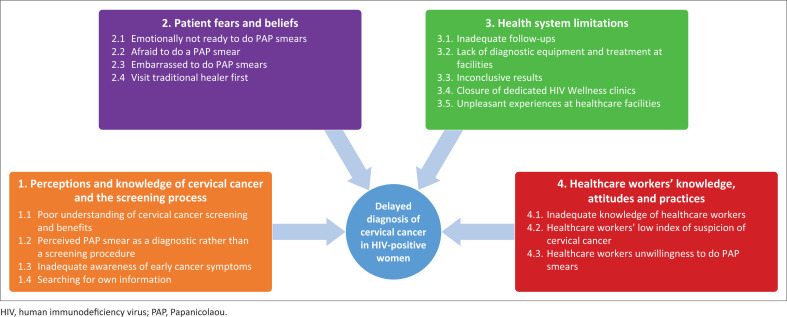
Illustration summarising themes and sub-themes.

Figure 1 represents the themes and their interrelatedness diagrammatically.

Theme 1 encompasses inadequate knowledge about the early warning signs of cervical cancer and the perception that PAP-smears are diagnostic rather than for screening as major barriers to cervical cancer screening. As a participant explained: ‘I thought it was normal because every month I bleed I thought my monthly cycle was changing, […] but I did not realize it was it [*cancer*]’ (Participant 8).

Theme 2 alluded to how personal beliefs and fears further delay cervical cancer screening. These included being emotionally not ready, procrastinating, as well as fears and embarrassment about doing the procedure. One participant explained:

‘I postponed many times because my heart was not willing to go, I would tell myself I am going tomorrow, but when the day comes, I would postpone and find an excuse not to go.’ (Participant 7)

Another explained how cultural beliefs also influence them:

‘The problem with tradition is that when you are not feeling well, they say you have been bewitched. I went there [to a traditional healer] before I got the results from the doctor.’ (Participant 5)

Theme 3 describes health system limitations that contribute to the delayed diagnosis. Poor follow-up arrangements, inadequate equipment, inconclusive PAP smear results, decentralisation of HIV clinics, and unpleasant experiences at facilities are all barriers to effective cervical cancer screening. As a participant mentioned:

‘I remember June last year I went to the clinic to do PAP smear, and they said they don’t have speculums. […] I’m not even sure if I’ll get help because they always sending us back. I just hope I’ll get help.’ (Participant 4)

Theme 4 emphasises how health workers’ inadequate knowledge and low index of suspicion influence their screening practices, resulting in delayed diagnoses. The unwillingness of healthcare workers further bars patients from getting PAP smears as required. This is highlighted by the quotation: ‘I came back many times, but they would give pills without doing anything. [*it*] felt like they are not giving me attention’ (Participant 4).

## Discussion

Our study highlights several factors contributing to the delay in the diagnosis of cervical cancer. Firstly, there is a misunderstanding that PAP smears are a diagnostic test rather than a screening tool necessary for the early identification of cervical cancer. Secondly, personal issues such as fear, anxiety, shyness, embarrassment, procrastination and a belief in traditional healers are barriers to screening practices. Thirdly, health system challenges such as unavailability of diagnostic consumables, broken equipment, competing health programmes, inconclusive results and inadequate treatment facilities exacerbate this. Fourthly, a low index of suspicion and apparent unwillingness of health workers to do cervical screening further delays the process.

The wrong information and perceptions patients had about cervical cancer screening led to delayed diagnosis of cervical cancer in HIV-positive women (theme 1). We found that, while women know about cervical cancer, they believed PAP smears were used to diagnose the condition rather than for early detection, so most did the test only when they were already unwell. Our study highlights those deficiencies in terms of patients’ understanding of the procedure itself led to delayed diagnosis. Some women poorly recognised the symptoms of cervical cancer and thought it was because of their HIV status. Similar factors have been identified elsewhere on the continent. Studies from Ethiopia, Tanzania, Botswana and KwaZulu-Natal confirm that a lack of knowledge about cervical cancer risk factors, early symptoms, and the frequency of screening, as well as the notion that PAP smears are diagnostic, all play a role in delayed diagnoses of cervical cancer.^13,14,15,16^

An interesting but concerning finding was that only one participant, with tertiary education, searched for her own information on the internet when she experienced unpleasant symptoms (see Table 2: theme 1.4). She was then able to seek appropriate care, unlike other participants who were less informed. For younger and educated participants, searching for medical information online has become an intrinsic part of modern medicine.^17^ It is therefore concerning that only one out of 10 participants in this study was empowered enough to do so.

**TABLE 2:** Main themes and sub-themes with supporting participant quotations.

Themes and sub-themes	Supporting quotations
**1. Perceptions and knowledge of cervical cancer and the screening process**	‘When they give us treatment, they do explain and they explain often. The nurses do help us. So when we go to the clinic to take our HIV treatment they do explain about other medical conditions. So I understand that I have to check for cervical cancer, because I don’t know what causes it’ (Participant 1)
1.1. Poor understanding of cervical cancer screening and benefits	‘I did not know what kind of a procedure it is and I was afraid to do it’, (Participant 9)
1.2. Perceived PAP smear as a diagnostic rather than a screening procedure	‘I felt that these pains are not normal and the only way to find out what was going on was to do PAP smear’ (Participant 4)
1.3. Inadequate awareness of early cancer symptoms	‘I thought it was normal because every month I bleed I thought my monthly cycle was changing, this month it can be at the end of the month for three days and the next month it can happen on the 15th, but I did not realize it was it [*cancer*]. I think the time I thought my cycle was changing I should have taken further steps and go to a doctor and not think it was my menstruation cycle’ (Participant 8)
1.4. Searching for own information	‘No, I do a lot of research, if I feel pain somewhere I take my phone and search what is going on, so that is why I forced to go to the clinic and also call someone who will help me, because if I just stay at home it will progress’ (Participant 4)
**2. Patient fears and beliefs**	
2.1. Emotionally not ready to do PAP smears	‘I postponed many times because my heart was not willing to go, I would tell myself I am going tomorrow, but when the day comes, I would postpone and find an excuse not to go, but I eventually forced myself to go because the situation was not getting better’ (Participant 7)
2.2. Afraid to do a PAP smear	‘They asked me to do it, but you know anxiety and fear, I was never free to do PAP smear. I did not know what kind of a procedure it is and I was afraid to do it. Have you seen that thing, now that I know, I see that is an easy thing to do, but I’m telling you this now, if it wasn’t for the fact that I had bleeding, I’m telling you, I would not have done it even now because of fear. I would even get palpitation when I thought of doing PAP smear’ (Participant 7)
2.3. Embarrassed to do PAP smears	‘Actually I don’t know what they are scared of because some of them when they talk they say they don’t want people to see them naked, very small kids are going to see us naked, Yes and also a person would say, I won’t agree to get tested by a specific person because they know me, so they feel like they will know things about them’ (Participant 6)
2.4. Visit a traditional healer first	‘The problem with tradition is that when you are not feeling well they say you have been bewitched. I went there before I got the results from the doctor. At home they took me there and the traditional doctor said they gave me something to eat that is bad, but because my father is a church goer he told me to follow all the instructions from the doctor and not to follow these people’s instructions’ (Participant 5)
**3. Health system limitations**	
3.1. Inadequate follow-ups	‘They didn’t give me a date for follow up, they said they will call me’ (Participant 1)
3.2. Lack of diagnostic equipment and treatment at facilities	‘So I remember June last year I went to the clinic to do PAP smear and they said they don’t have speculums. Sometimes they will send you back and tell you the equipment is not working, and the sickness gets worse. That’s very important because we go early but you will go back home without help. I was supposed to go for radiation from April I’m only going now and I’m not even sure if I’ll get help because they always sending us back. I just hope I’ll get help’ (Participant 4)
3.3. Inconclusive results	‘I did PAP smear then it came back and had another PAP smear then it came back and had another PAP smear. Then when it came back, they say they are not seeing clearly, so I had 4 PAP smears. I don’t know because I was not told what the problem was, I was only told that it’s complicated and that they are not okay’ (Participant 2)
3.4. Closure of dedicated HIV Wellness clinics	‘In the previous years we did do PAP smear while taking treatment from wellness, every year we would do PAP smear. So ever since we stopped going to wellness and where referred us to clinics, we are not doing them routinely’ (Participant 6)
3.5. Unpleasant experiences at healthcare facilities	‘But I’m not happy because the first time when they cut me, I never healed, the stiches made me struggle. I couldn’t walk. They gave me medication, but I was not getting better. I came back many times, but they would give me sutures and pills without doing anything’ (Participant 4)
**4. Healthcare workers’ knowledge, attitudes and practices**	
4.1. Inadequate knowledge of healthcare workers	‘When I consulted with the doctor, he said what he is seeing he has not seen it before so he can’t say what type of thing it is. Then he said he will call another doctor then the second doctor said it has affected the skin on the outside’ (Participant 6)
4.2. Healthcare workers’ low index of suspicion of cervical cancer	‘I went to the clinic because I had pains on the left side below the belly button. So, when I got to the clinic, they gave me pills and I was told that I have an infection. But I went back with the same problem, but they still said it’s an infection’ (Participant 6)
4.3. Healthcare workers unwillingness to do PAP smears	‘I came back many times, but they would give pills without doing anything. So, I went back until I called someone I know at the clinic and said to them that I came there twice trying to do PAP smear and felt like they are not giving me attention’ (Participant 4)

HIV, human immunodeficiency virus; PAP, Papanicolaou.

Several barriers to internet searching of health information in Africa have previously been identified, including a lack of appropriate devices, inadequate internet access, limited skills and understanding.^18^ This is a phenomenon that needs more research in our setting.

Our participants highlighted several personal barriers to going for PAP smears (theme 2), including not being ready or being afraid to do the test, procrastination, being embarrassed and shy to undress in front of healthcare workers. Researchers in Botswana and Kenya also found that a lack of privacy and perceived violation of privacy were barriers to early screening for cervical cancer.^12,18^ As in our study, patients in KwaZulu-Natal frequently go to traditional healers before consulting at health facilities, leading to delays in diagnosing cervical cancer.^14^

Healthcare system deficiencies, such as poor follow-up arrangements and limited resources to do PAP smears, were mentioned as barriers to the diagnosis of cervical cancer (theme 3). These limitations in the healthcare system have been mentioned in the National cervical cancer screening guidelines as well as in studies carried outin Malawi and Botswana.^8,13,20^ Negative experiences at health facilities influence participants’ health-seeking behaviour and their confidence in the health system to help them (theme 3). The negative consequences of unpleasant experiences at health facilities were also described in studies performed in Botswana, Malawi and Uganda.^7,13,20^ We found that inconclusive results, repeating the tests without adequate explanation, uncertainty expressed by healthcare workers and unpleasant treatment interventions frustrated participants (themes 3.3, 3.5 and 4.1).

Some of our participants felt that when HIV services were decentralised and moved from the hospital to primary care clinics, it led to a reduction in PAP smear screening for women living with HIV (theme 3.4). Studies from Nigeria and Tanzania found the opposite, suggesting that decentralisation allows for more screening.^6,16,21^ Adepuju et al. suggested in the Nigerian study that so-called ‘more important’ services, such as maternal health, compete negatively with cervical cancer screening.^21^

Healthcare worker attitudes and their competence were viewed as some of the main reasons for the delayed diagnosis of cervical cancer (theme 4). Our findings indicate that some participants perceived healthcare workers as being unwilling or not interested in doing cervical cancer screening (theme 4.3). Three South African qualitative studies among nurses and patients and a KAP study among Saudi Arabian nurses, echoed this reluctance among health workers, who claimed cultural taboos and limited resources as barriers to cervical cancer screening.^5,10,14,22^ These findings are concerning, considering that primary healthcare clinics are the source of information for most patients about appropriate and recommended screening practices.

### Limitations

The study was conducted at one hospital in Limpopo with only 10 participants, who were HIV-positive, meaning that the findings may not be transferable to all settings. No clear data saturation point was reached as several of the identified possible participants were unable to participate in the study. This could have skewed the results. Answers that were socially acceptable and memory limitations could have further contributed to bias.

Another limitation of the study is that a male research assistant conducted the interviews, which could have hindered the participants from talking freely about what is perceived as women’s matters. Although the research assistant was well versed in doing qualitative interviews, the researcher was fairly novice in the field, which could have added to bias.

## Conclusion

Four major factors appear to contribute to delays in cervical cancer diagnoses in HIV-positive women in a rural hospital in Limpopo. These included that patients perceived PAP smears to be diagnostic rather than for screening purposes, and that they had limited knowledge about the frequency of PAP smear screening. In addition, patients’ fears and beliefs contributed to delays. Healthcare system limitations also played a role. Finally, health workers’ limited knowledge, low index of suspicion and unwillingness to do PAP smears prevented patients from receiving a timely diagnosis.

Health education on cervical cancer prevention strategies, adequate resources, monitoring of screening practices and the motivation of health workers, coupled with a patient-centred approach, are needed to ensure adequate screening for cervical cancer in HIV-positive women. Further research on empowering patients from rural settings to search for their own health information may be crucial to combat the high burden of cervical cancer in HIV-positive women in southern Africa.
